# Congenital Anomalies of the Pancreas: Various Clinical Manifestations and Their Impact on Pancreatic Diseases and Outcomes

**DOI:** 10.7759/cureus.27915

**Published:** 2022-08-12

**Authors:** Sharath Kumar V, Prashanth Sangu, Kolandasamy C, Prabhakaran R, Sugumar Chidambaranathan, Naganath Babu Obla Lakshmanamoorthy

**Affiliations:** 1 Surgical Gastroenterology, Madras Medical College, Chennai, IND

**Keywords:** ectopic pancreas, annular pancreas, pancreatic divisum, dorsal agenesis, congenital anomaly

## Abstract

Background and objective: Congenital anomalies of the pancreas are relatively uncommon. Most of these are asymptomatic and are detected incidentally, but can present with a variety of clinical manifestations like pancreatitis, duodenal obstruction, biliary obstruction, and rarely malignancy. Here in our study, we describe various congenital anomalies of the pancreas associated with various clinical manifestations, its management strategies, and outcomes. The aim was to study the various clinical manifestations of and management strategies for pancreatic diseases associated with congenital anomalies of the pancreas and their outcomes.

Methods: A retrospective analysis of a prospectively maintained institutional database of 14 patients, admitted over a period of three years from June 2019 to May 2022, who were treated for different clinical manifestations of various congenital anomalies of the pancreas and their outcomes was done at our institution.

Results: The total number of congenital anomalies of the pancreas in our study was 14 out of whom 7 (50%) were males and 7 (50%) females. The mean age of the patients was 37 years. The most common congenital anomaly was pancreatic divisum in six (42.9%) cases. The most common clinical manifestation was acute pancreatitis in four (28.6%) cases. One (7.1%) case was incidentally detected intraoperatively for another condition. Eight (57.1%) patients underwent surgical intervention and six (42.9%) patients were medically managed. Mortality occurred in two (14.3%) cases. Associated alcohol consumption was seen in 2 (14.3%) cases; 10 (71.4%) patients had no comorbidities while 4 (28.6%) patients had diabetes mellitus. Out of eight (57.1%) surgical patients, two (25%) had Clavien-Dindo grade I and one patient (12.5%) grade V complications.

Conclusion: Congenital anomalies of the pancreas can be associated with a variety of clinical manifestations; their management strategies and outcomes are no different from patients with the same clinical manifestations with normal pancreatic development.

## Introduction

Congenital anomalies of the pancreas arise from the failure of complete rotation and fusion during embryogenesis [1]. The most common congenital anomaly is pancreatic divisum [1,2]. Other anomalies include the annular pancreas, dorsal agenesis of the pancreas, complete agenesis, and ectopic pancreas [1,2]. Most of these congenital conditions go undetected until adulthood when the patient comes to medical attention with nonspecific symptoms or is discovered incidentally [3]. These patients can also present with various clinical manifestations such as acute and chronic pancreatitis, duodenal obstruction, biliary obstruction, and rarely malignancy [3]. In this study, we describe various congenital anomalies of the pancreas with associated clinical manifestations, and their management strategies and outcomes.

## Materials and methods

The study was conducted at the Institute of Surgical Gastroenterology, Madras Medical College and Rajiv Gandhi Government General Hospital, Chennai, based on a retrospective analysis of a prospectively maintained institutional database of 14 patients diagnosed with various congenital anomalies of the pancreas with associated clinical manifestations, admitted over three years from June 2019 to May 2022. The medical records of these patients were retrieved and various details including demographics, presentation, management, comorbidities, complications, and any deviation from the usual management strategies for pancreatic diseases were determined.

A literature search was performed on the PubMed database for relevant articles on the congenital anomalies of the pancreas. Only full-text articles published in the English language were included. Studies that reported different clinical presentations, management, and complications were studied.

Inclusion and exclusion criteria

Patients with all types of congenital anomalies of the pancreas with various clinical manifestations who presented to our center and received treatment were included in the study. Asymptomatic patients with incidentally detected congenital anomalies of the pancreas in imaging evaluation for other conditions were excluded.

Diagnosis

Diagnosis was made based on the clinical history, laboratory findings such as complete blood picture, liver function test, serum amylase, serum lipase, and radiological imaging in the form of ultrasound, abdomen contrast-enhanced computed tomography (CECT), and abdomen magnetic resonance cholangiopancreatography (MRCP).

Statistical analysis

Descriptive statistical analyses were carried out in the present study. Results on continuous measurements were presented as means ± SDs and results on categorical measurements were presented in number (%). Microsoft Word and Excel (Microsoft, Redmond, Washington) were used to generate tables.

## Results

The total number of congenital anomalies of the pancreas in our study was 14 over the three-year period; out of them, seven (50%) were males and seven (50%) females (Table 1). The mean age of the patients was 37 years (ranging from 13 to 72 years). In our series, the most common congenital anomaly was pancreatic divisum in six (42.9%) cases followed by dorsal agenesis of the pancreas in four (28.6%) cases, annular pancreas in two (14.3%), and one (7.1%) case of ectopic pancreas and situs inversus each. The most common clinical manifestation is acute pancreatitis in four (28.6%) cases followed by chronic pancreatitis in two (14.3%) cases, malignancy in two (14.3%) cases, pancreatic type of pain in one (7.1%), cholelithiasis in one (7.1%), chronic pancreatitis with cholelithiasis in one (7.1%), duodenal obstruction in one (7.1%) and acute pancreatitis with duodenal obstruction and jaundice in one case (7.1%) (Table 2). One (7.1%) case of the ectopic pancreas was incidentally detected intraoperatively while operating for type IV Mirizzi syndrome. Eight (57.1%) patients underwent surgical intervention and six (42.9%) patients were medically managed. Mortality occurred in two (14.3%). Associated alcohol consumption was seen in two (14.3%) cases; 10 (71.4%) patients had no comorbidities while four (28.6%) had diabetes mellitus (one patient had associated alcoholic liver disease). All patients underwent abdominal ultrasound and CECT for initial imaging diagnosis and findings were confirmed by MRI with MRCP. Postoperative complications were graded according to the Clavien-Dindo grade. Out of eight (57.1%) surgical patients, two patients (25%) had Clavien-Dindo grade I and one patient (12.5%) had grade V complications.

**Table 1:** Congenital anomalies of the pancreas (n=14) n: total number of patients

Diagnosis	Number	Percentage
Pancreatic divisum	6	42.9
Dorsal agenesis of the pancreas	4	28.6
Annular pancreas	2	14.3
Ectopic pancreas	1	7.1
Situs Inversus	1	7.1

**Table 2:** Clinical manifestations in patients with congenital anomalies of the pancreas (n=14)

Clinical manifestation	Number	Percentage
Acute pancreatitis	4	28.6
Chronic calcific pancreatitis	2	14.3
Duodenal obstruction	1	7.1
Acute pancreatitis + duodenal obstruction + biliary obstruction	1	7.1
Pancreatic type pain	1	7.1
Malignancy	2	14.3
Cholelithiasis	1	7.1
Cholelithiasis + chronic calcific pancreatitis	1	7.1
Incidental findings during surgery	1	7.1

Pancreatic divisum

**Figure 1:**
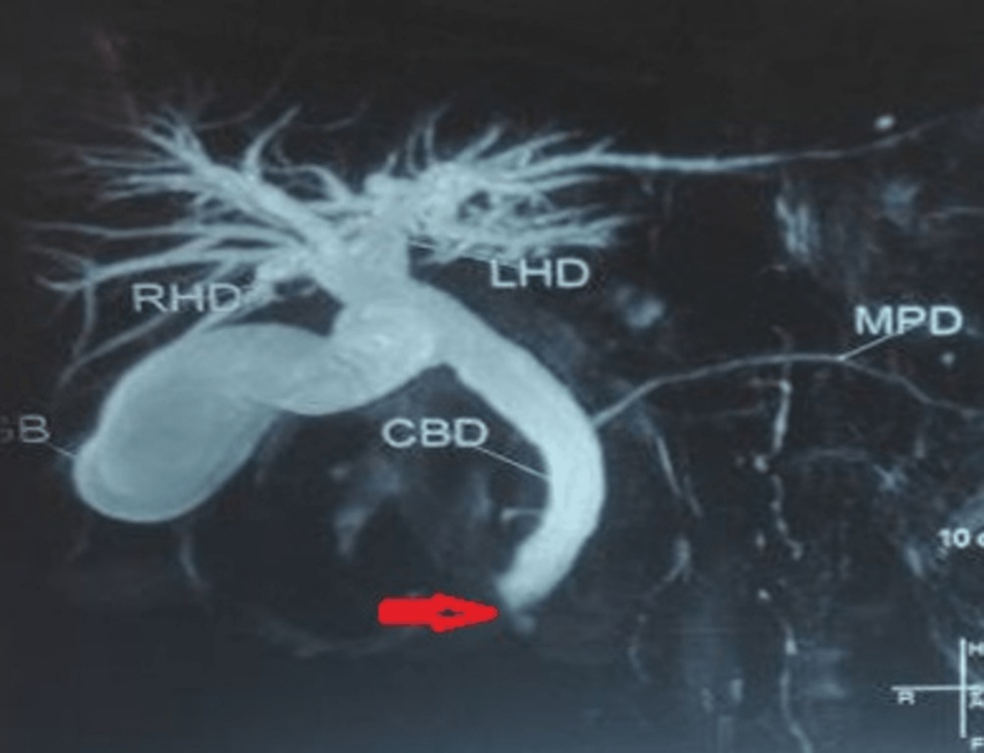
MRCP shows an abrupt cut-off of distal CBD (red arrow) due to the periampullary growth in a patient with PD MRCP: magnetic resonance cholangiopancreatography; CBD: common bile duct; PD: pancreatic divisum

Out of six (n=14) cases of pancreatic divisum, the most common clinical manifestation was chronic calcific pancreatitis in three (50%) cases (one case associated with cholelithiasis) and one case each (16.5%) of acute mild pancreatitis, biliary colic, and periampullary carcinoma. The most common clinical presentation was abdominal pain in five (83%) cases and one (17%) with obstructive jaundice (Table 3). Out of three patients with chronic calcific pancreatitis, two patients were treated surgically with Frey’s procedure (cholecystectomy in addition to Frey’s procedure in one of the cases due to associated cholelithiasis) and one with medical management. One patient with an index case of acute mild pancreatitis was managed conservatively with medications. One patient with biliary colic due to gall stone underwent laparoscopic cholecystectomy. One patient with periampullary carcinoma underwent Whipple’s procedure with modified Buchler’s technique (Figure 1) and this patient succumbed to myocardial infarction on postoperative day 10. There was no alcohol intake history in all of these patients.

**Table 3:** Clinical manifestation, presenting symptoms, and management of pancreatic divisum (n=14)

Case	Age	Sex	Clinical manifestation	Presenting symptom	Comorbidities	Management	Postoperative complications (Clavien-Dindo grade)
1	17	F	Acute mild pancreatitis	Pain	-	Medical	-
2	13	F	Biliary colic	Pain	-	Laparoscopic cholecystectomy	Grade 0
3	18	F	Chronic calcific pancreatitis	Pain	Diabetes mellitus	Frey’s procedure	Grade 0
4	45	M	Chronic calcific pancreatitis + Cholelithiasis	Pain	-	Frey’s procedure + laparoscopic cholecystectomy	Wound infection (Grade I)
5	21	F	Chronic calcific pancreatitis	Pain	-	Medical	-
6	72	M	Periampullary carcinoma	Jaundice	-	Whipple’s procedure	Death due to a cardiac event (Grade V)

Dorsal agenesis of pancreas

**Figure 2:**
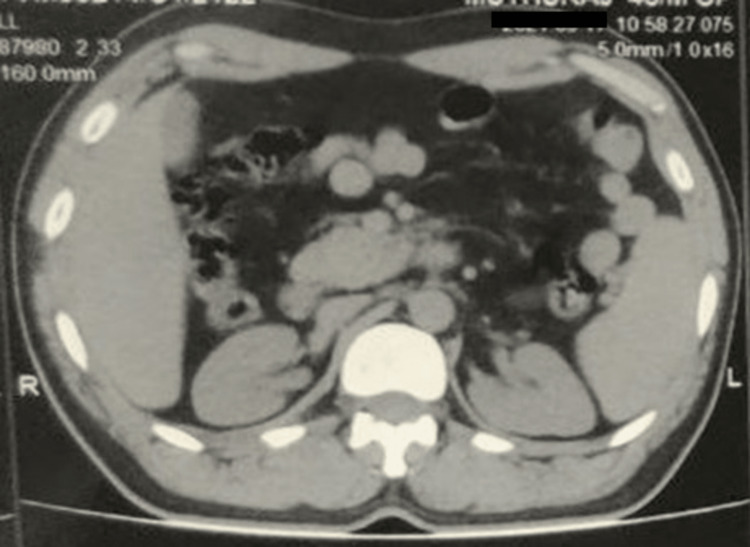
Computed tomography shows dorsal agenesis in a patient with acute mild pancreatitis

**Figure 3:**
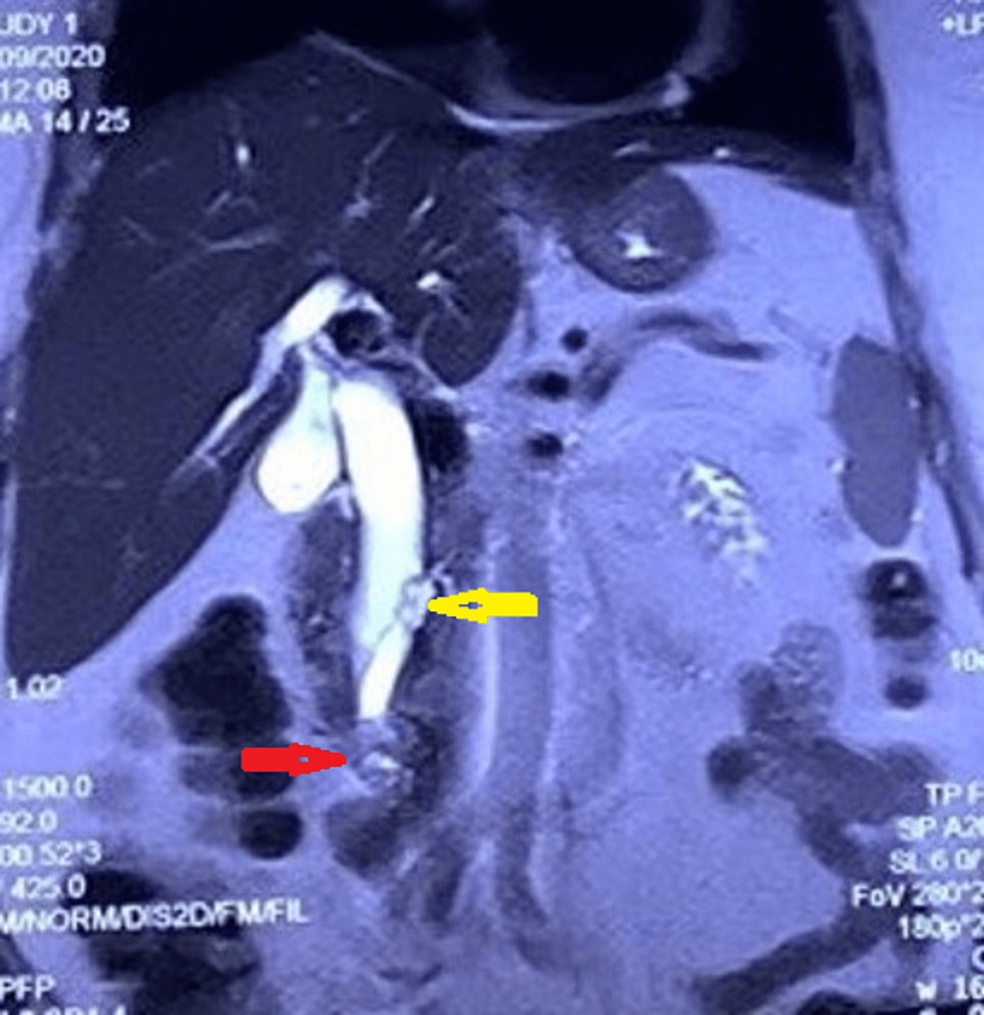
Magnetic resonance imaging shows an absent pancreatic duct in the body and tail of the pancreas (yellow arrow) with a mass lesion (red arrow) at the distal common bile duct

**Figure 4:**
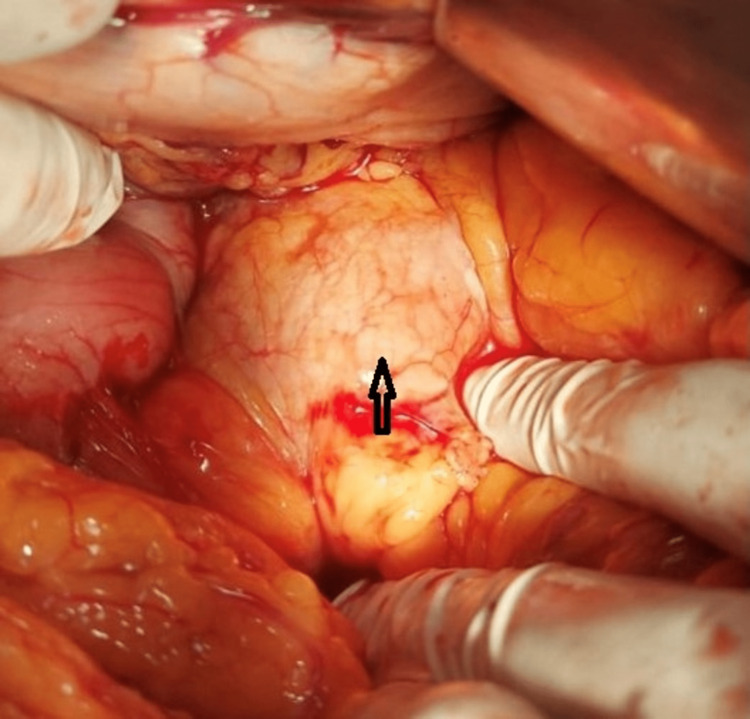
Intraoperative image shows a mass in the head of the pancreas (black arrow) in a patient with dorsal agenesis of the pancreas

Out of four patients (28.6%) with dorsal agenesis of the pancreas, two (50%) had complete and two (50%) had incomplete agenesis (Table 4). The most common presentation was acute mild pancreatitis in two (50%) patients and pancreatic type of pain in one (25%) that was managed with medical treatment (Figure 2). One (25%) patient who presented with obstructive jaundice and diabetic ketoacidosis had a periampullary growth. CT and MRI showed a hypodense mass lesion of size 1.9 x 1.3 cm in the head of the pancreas with an absent body and tail of the pancreas (Figure 3). Intraoperatively, there was a mass in the head of the pancreas along with the absent body and tail of pancreas (Figure 4). Fine needle aspiration cytology (FNAC) of the mass lesion was suggestive of mixed carcinoma (acinar cell and ductal carcinoma). The patient underwent biliodigestive bypass followed by palliative chemotherapy due to poor general condition. All four patients were non-alcoholic and two (50%) of them had diabetes mellitus.

**Table 4:** Clinical manifestation, presenting symptoms, and management of the dorsal agenesis of the pancreas (n=14)

Case	Age	Sex	Clinical manifestation	Presenting symptom	Comorbidities	Management	Postoperative complications (Clavien-Dindo grade)
1	44	F	Acute mild pancreatitis	Pain	Diabetes	Medical	-
2	46	M	Acute mild pancreatitis	Pain	-	Medical	-
3	19	F	Pancreatic type of pain	Pain	-	Medical	-
4	62	F	Periampullary carcinoma	Pain, jaundice	Diabetes	Biliodigestive bypass and palliative chemotherapy	Grade 0

Annular pancreas

**Figure 5:**
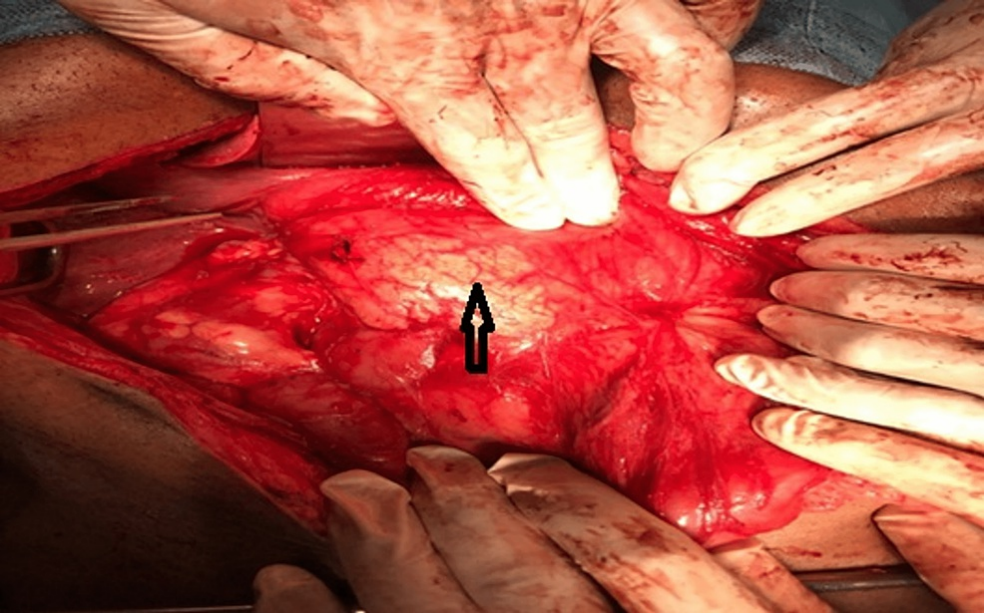
Intraoperative image of the complete annular pancreas (black arrow)

Out of the total 14 cases, two (14.3%) patients had an annular pancreas (Table 5). One patient had a complete annular pancreas causing duodenal obstruction with abdominal pain and vomiting as a prominent feature, and underwent surgical management with gastrojejunostomy (Figure 5). Another patient with known diabetic and alcoholic liver disease who had an incomplete annular pancreas presented with acute severe pancreatitis with duodenal and biliary obstruction and succumbed to multi-organ dysfunction syndrome.

**Table 5:** Clinical manifestation, presenting symptoms, and management of annular pancreas (n=14)

Case	Age	Sex	Clinical manifestation	Presenting symptom	Comorbidities	Management	Postoperative complications (Clavien-Dindo grade)
1	59	M	Duodenal obstruction	Vomiting, pain	-	Gastrojejunostomy	Grade 0
2	42	M	Acute severe pancreatitis, duodenal and biliary obstruction	Vomiting, pain, jaundice	Diabetes mellitus, alcoholic liver disease	Medical	-

Ectopic pancreas

**Figure 6:**
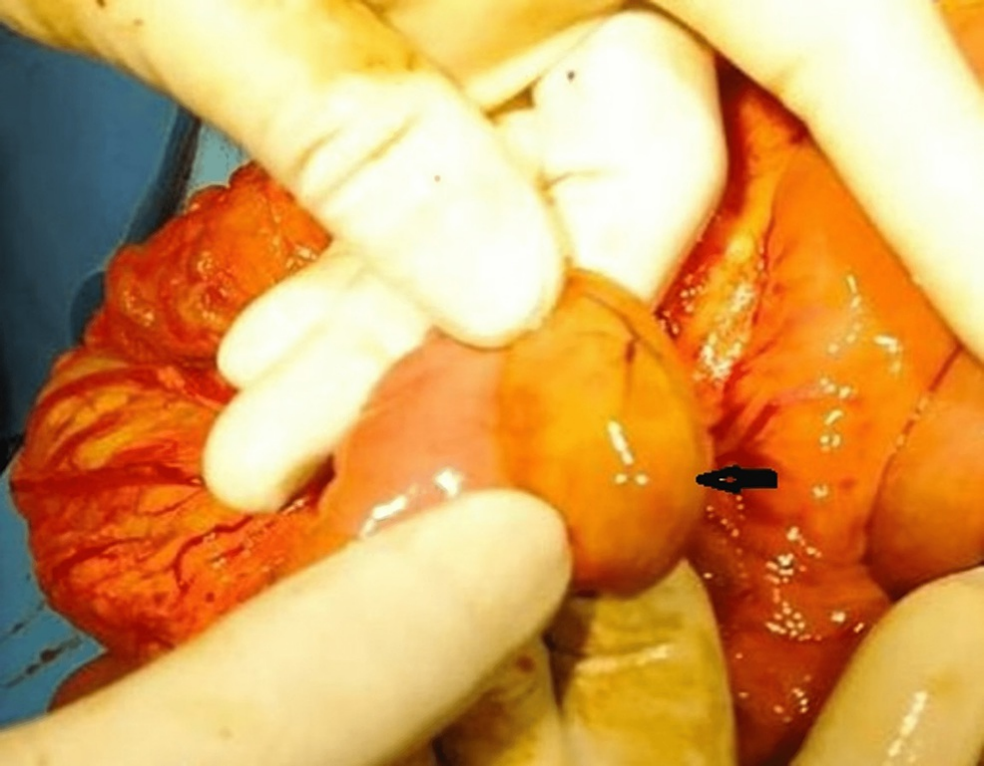
Intraoperative image of the ectopic pancreas (black arrow) in the proximal jejunum

**Figure 7:**
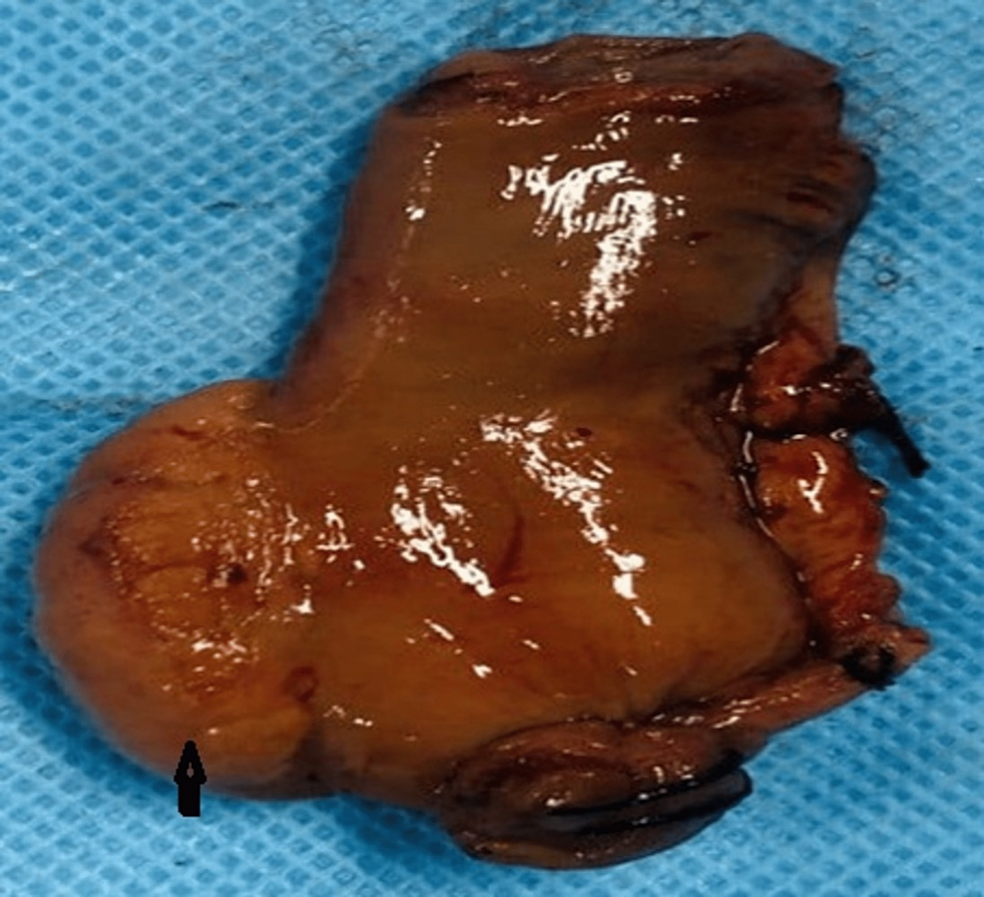
Ectopic pancreas specimen

One case presented with abdominal pain and jaundice with the ectopic pancreas incidentally detected in the proximal jejunum during surgery for type IV Mirizzi syndrome, for which we did cholecystectomy with Roux-en-Y hepaticojejunostomy (Figure 6). We did jejunal segmental resection containing the ectopic pancreas (Figure 7). Postoperative recovery was uneventful except for wound infection (Clavien-Dindo grade I).

Situs inversus

**Figure 8:**
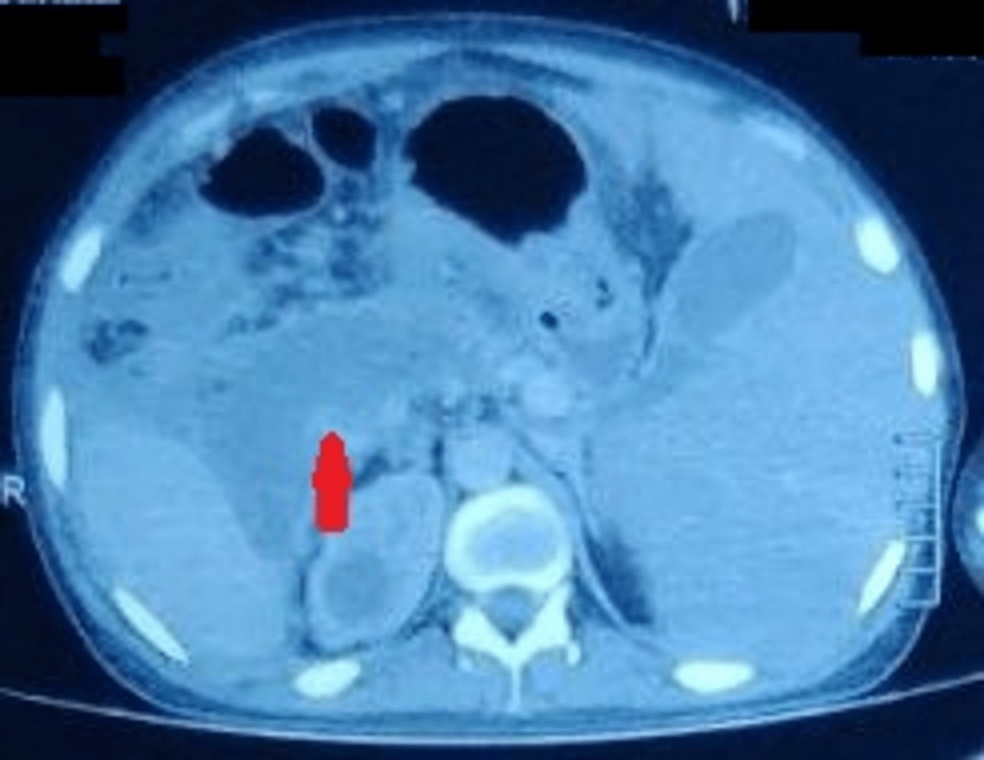
Computed tomography of the abdomen showing situs inversus with necrotic collection (red arrow) in the pancreas

One patient who was a chronic alcoholic presented with acute necrotizing pancreatitis and was found to have situs inversus when evaluated with abdomen CT (Figure 8). He underwent a right retroperitoneal necrosectomy after not responding to the step-up approach with multiple percutaneous interventions for necrotic collection; the patient later recovered.

## Discussion

Congenital anomalies of the pancreas arise from the failure of complete rotation and fusion during embryogenesis [3]. Pancreas divisum is the most common congenital anomaly of the pancreas that results from an abnormal fusion of the ventral and dorsal pancreatic ducts during fetal development with a reported incidence of 3.6% to 5.8% [3,4]. A complete pancreas divisum is defined as a completely separate pancreatic duct system. An incomplete pancreas divisum is an anatomic variation that has inadequate communication, usually an extremely small branch, between the ventral and dorsal pancreatic ducts.

Pancreatic divisum is found in up to 25% of patients with acute idiopathic pancreatitis; however, up to 5% can be expected to develop symptoms [4]. In our study, one patient had acute mild pancreatitis (16.5%) in the pancreatic divisum group. Out of six cases of pancreatic divisum in our study, two (33%) patients were medically managed and four (67%) patients underwent surgical management. Pancreas divisum represents a duct anomaly in the pancreatic head ducts, frequently leading to recurrent acute pancreatitis or chronic pancreatitis [5]. Dorsal duct hypertension secondary to an inadequate flow through a narrowed segment of the duct of Santorini or the papilla leads to chronic pancreatitis and recurrent acute pancreatitis [5]. Frey’s procedure is a safe and efficacious procedure for the relief of the complications of recurrent acute pancreatitis and chronic pancreatitis in patients with pancreatic divisum [6]. In our study, two (33%) cases of pancreatic divisum with chronic calcific pancreatitis underwent Frey’s procedure.

The frequency of pancreatic divisum was also found to be significantly increased in patients with gallbladder stones [7]. In a retrospective series, by Bernard et al., of 1,825 successful consecutive endoscopic retrograde cholangiopancreatographies, 137 cases (7.5%) of pancreas divisum were found and this anomaly was associated with acute biliary pancreatitis in 23.7% of patients [7]. In our study, two patients had associated cholelithiasis.

In a study by Takuma et al., pancreatic carcinoma occurred in 5 (9.2%) of 54 patients with pancreatic divisum, and all carcinomas occurred in the dorsal pancreas [8]. In a study by Okada et al., pancreatic carcinoma developed in the dorsal pancreas in 24 of 25 cases of pancreatic carcinoma associated with pancreas divisum [9]. Nishino et al. also reported that pancreatic carcinoma developed in the dorsal pancreas in 11 of 12 cases with pancreas divisum [10]. Long-standing dorsal duct obstruction caused by the relative stenosis of the minor duodenal papilla might be a factor that promotes pancreatic carcinoma [11,12].

Gregório et al. described a case of synchronous periampullary tumor in a patient with pancreatic divisum and neurofibromatosis type 1 [13]. In an analysis of MRCP by Adibelli et al., 90 out of 1628 patients were found to have pancreatic divisum; out of them 7.8% had pancreaticobiliary tumors including intrapancreatic mucinous neoplasm, ampullary carcinoma, pancreas carcinoma, and gallbladder carcinoma [14]. In our study, one patient with pancreatic divisum had periampullary carcinoma and underwent Whipple’s procedure.

Agenesis of the dorsal pancreas is a rare congenital anomaly that results from the embryological failure of the dorsal pancreatic bud to form the body and tail of the pancreas. In partial agenesis of the dorsal pancreas, the minor papilla, duct of Santorini or the pancreatic body are present. In complete agenesis of the dorsal pancreas, the neck, the body, and the tail of the pancreas, the duct of Santorini, and minor papilla are absent [15]. In our study, two (50%) patients had a partial and the other two (50%) had complete agenesis. The first case of dorsal agenesis was described in 1911 in an autopsy finding, and so far, around 100 cases have been reported in the world scientific literature till the year 2020 [16-18]. Pancreatitis is a commonly associated disease in patients with agenesis of the dorsal pancreas [18]. In our series, two patients (50%) had mild acute pancreatitis and one patient (25%) had the pancreatic type of pain, all managed medically. Approximately 50% of patients with dorsal agenesis of pancreas also have concomitant hyperglycemia [18,19]. In our study, two (50%) patients had diabetes mellitus.

To our knowledge, to date, only 17 cases of pancreatic tumors are among the very few reported cases of dorsal agenesis of pancreas in the world literature [20]. Among them, the most common is ductal adenocarcinoma, as found in 12 cases, followed by two cases of neuroendocrine tumors and three precancerous [20]. In our study, one patient (25%) with dorsal agenesis of the pancreas had mixed carcinoma (acinar cell and ductal carcinoma), and this is the first case of mixed acinar ductal carcinoma reported in the literature, to our knowledge.

The annular pancreas is a rare congenital anomaly that results from the failure of the ventral bud to rotate with the duodenum, resulting in the envelopment of the rim of pancreatic tissue around the second part of the duodenum [21]. The rim of pancreatic tissue encircles partially (incomplete annular pancreas) or completely (complete annular pancreas) the second part of the duodenum, with the reported incidence in adults varying from 0.005% to 0.015% [22,23].

In a study by Zyromski et al., most common presentations of the annular pancreas were abdominal pain (75%) followed by gastrointestinal symptoms like vomiting (24%), pancreatitis (22%), and obstructive jaundice (6%) [24]. In our study, one patient who had a complete annular pancreas presented with abdominal pain and vomiting, and another with an incomplete annular pancreas presented with acute severe pancreatitis, vomiting, and jaundice.

The ectopic pancreas is a normal pancreatic tissue located at an abnormal location that lacks anatomic or vascular continuity with the main gland. It is usually asymptomatic, but may become symptomatic when complicated by pathologic changes such as inflammation, bleeding, obstruction, or malignancy. Prevalence ranges from 0.6% to 13.7% in autopsy series [25-27]. The ectopic pancreas is resected even when asymptomatic because the diagnosis cannot be made clinically and the main differential diagnosis is malignancy [27]. In our study, in one case, ectopic pancreas located in the proximal jejunum was found incidentally during another operation and we did segmental resection of the jejunum containing the ectopic pancreas. One patient with acute necrotizing pancreatitis was found to have situs inversus when evaluated with abdomen CT and underwent right retroperitoneal necrosectomy. Till now, to our knowledge, there is only one case report available in the literature on acute pancreatitis in a patient with situs inversus/polysplenia syndrome [28].

The present study also had some limitations. This was a retrospective study, and the sample size was small due to the rare occurrence of congenital anomalies of the pancreas. Only a few case reports and a series of individual anomalies of the pancreas are reported in the literature.

## Conclusions

Our study shows that congenital anomalies of the pancreas can present with a variety of clinical manifestations that may require appropriate treatment strategy. One should have a high index of suspicion if there are persistent and unexplained signs and symptoms such as abdominal pain, nausea, vomiting, and recurrent pancreatitis. Patients should be evaluated for congenital anomalies of the pancreas if no other cause is found. Management strategies and outcomes in patients treated for various clinical manifestations of congenital anomalies of the pancreas are no different from patients with the same clinical manifestations with normal pancreatic development.
